# Evaluating the dynamics of fee-free higher education in South Africa: a causal loop diagram approach 

**DOI:** 10.12688/f1000research.152478.3

**Published:** 2025-01-20

**Authors:** Tlotlo Ramasu, Grace Kanakana-Katumba

**Affiliations:** 1 Industrial Engineering, Tshwane University of Technology, Pretoria, Gauteng, 0183, South Africa

**Keywords:** Fee-free education, Higher education, Inclusive growth, Education equity, Socioeconomic progress, Causal loop diagran

## Abstract

**Background:**

This research investigated the dynamics of fee-free higher education in South Africa, analysing the interplay of socioeconomic, political, and institutional factors to elucidate the complexities surrounding its implementation and impact. Using a causal loop diagram approach (CLD), the study aims to identify key factors to effective implementation, how they interact to shape the outcome of the policy, and actionable strategies to address the challenges hindering the policy sustainability.

**Method:**

A participatory approach was used to developing the CLD, integrating insights from an extensive literature review and stakeholder opinion. The initial CLD was constructed based on evidence from prior research contextualizing fee-free higher education within South Africa’s unique socio-political sector. Discussions with stakeholders and experts provided critical feedback to refine the model, ensuring its relevance and accuracy. The CLD methodology was selected for its ability to illustrate complex interdependencies and systemic challenges.

**Results:**

Key findings highlight the impact of funding constraints, which create reinforcing feedback loops limiting access and infrastructure improvements. Also, administrative inefficiencies within the National Student Financial Aid Scheme (NSFAS) result in delays and inconsistencies, reducing policy inclusivity, and decentralized implementation across institutions perpetuates disparities in quality and access.

**Conclusion:**

The study concludes the urgent need for systemic reforms, including digitising financial aid processes, centralising NSFAS operations, and improving resource allocation to under-resourced institutions. Furthermore, the study aligns with global goals, particularly Sustainable Development Goals (SDGs) 4 (Quality Education), 8 (Decent Work and Economic Growth), and 10 (Reduced Inequalities), as well as the African Agenda 2063’s focus on human capital development. While the findings offer valuable recommendations for policymakers, further research is needed to explore longitudinal impacts of these reforms and expand comparative analyses with other nations implementing similar policies.

## 1. Introduction

Higher education in South Africa is pivotal in shaping the nation’s socioeconomic sector and fostering individual and national development ( Leibowitz & Bozalek, 2014). Spreen & Vally (2006) point out that since the end of apartheid in 1994, South Africa has been actively expanding access to tertiary education, aiming to address historical disparities and promote inclusivity. The country boasts a diverse higher education sector comprising universities, universities of technology, and comprehensive institutions ( Lange, 2020), offering a wide array of programs spanning various disciplines to cater to the diverse needs of students and industries alike. However, as Yende & Mthombeni (2023) stated, financial barriers have long hindered access to higher education, particularly for disadvantaged communities. Historically, tuition, accommodation, and related expenses have presented a significant obstacle for many aspiring students, perpetuating socioeconomic inequalities and limiting the realisation of individual potential ( Mutekwe, 2017). The significance of this study lies in its use of a methodology capable of analyzing the causality of relationships involved in the complex discourse on fee-free higher education through the development of a participatory causal loop diagram. This is aimed at assisting policymakers come to an informed decision on how the interrelationship between these variables will be affected by their decision on the subject of fee-free higher education.

As denoted by Kioupi & Voulvoulis (2019), education is a cornerstone for national progress. In the context of South Africa, characterised by its status as a developing nation, a pressing need exists for a skilled workforce ( Vogel, 2015). The country grapples with staggering levels of income inequality, evidenced by a Gini coefficient of 0.67, the highest globally ( Vogel, 2015). According to Barro (2013), education emerges as a potential avenue to mitigate the stark disparities between affluent and marginalised communities. Additionally, South Africa contends with a formidable unemployment rate of 31.9%, according to ( Lauder & Mayhew, 2020). Many unemployed individuals lack the requisite skills for gainful employment, underscoring the pivotal role of education in enhancing their employability ( Lauder & Mayhew, 2020).

Post-apartheid South Africa’s educational policy has focused on equitable access to higher education to promote social mobility and economic development ( Ayuk & Koma, 2019; Varghese et al., 2023). A significant initiative in this regard is fee-free higher education for eligible students, introduced following the #FeesMustFall protests in 2015, which highlighted the systemic failures in serving marginalized communities ( Ntombana et al., 2023; Akala, 2023). President Jacob Zuma’s 2017 announcement of tuition-free education aimed to alleviate financial barriers and promote inclusivity ( Spreen & Vally, 2006). The dynamics of this policy involve various stakeholders and aim to address historical disparities ( Mokgotho et al., 2023).

In response to these challenges, the South African government has implemented various policies and initiatives to democratise access to higher education ( Salmi & D’Addio, 2021). Following the study by Salmi & D’Addio (2021), one such initiative is the introduction of fee-free higher education for eligible students, which seeks to alleviate financial burdens and enhance inclusivity within the tertiary education sector. However, this initiative exists within a complex system of interconnected socioeconomic, political, and institutional factors, each influencing its implementation and outcomes ( Mlambo et al., 2018). This study is guided by complexity science, which views policy environments as dynamic systems characterised by interdependent variables, feedback loops, and emergent behaviours, as described by McDonald (2009). This framework recognises that policy outcomes are shaped not only by direct interventions but also by the interactions and interdependencies within the broader system. Within this context, the research employs causal loop diagrams (CLDs) as a methodological tool to analyse the dynamics of fee-free higher education in South Africa, as supported by Crabolu et al. (2023). The research by Forrester (2009) also stated that CLDs are grounded in systems thinking, providing a visual representation of causal relationships and feedback loops among key factors. This approach aligns with existing literature on educational policy analysis, which increasingly emphasises the importance of understanding education policies as complex systems rather than isolated interventions, as emphasised by Faham et al. (2017).

In addition, by mapping out causal relationships and feedback loops, causal loop diagrams facilitate a systemic understanding of fee-free higher education dynamics. This holistic perspective helps researchers uncover underlying patterns and mechanisms that drive policy implementation, impact, and outcomes, thereby informing more effective intervention strategies, as Abidin et al. (2017) highlighted.

Furthermore, unlike quantitative modelling techniques, such as econometric or statistical models, causal loop diagrams prioritise qualitative analysis and conceptual mapping. This approach is well-suited to exploratory research endeavours seeking to elucidate the complexities of social systems and policy environments, where precise numerical data may be limited or uncertain, as emphasised by Haraldsson (2004). Causal loop diagrams offer a participatory research tool that can engage stakeholders in the research process, similar to the research methodology ( Toole, 2005). Researchers can harness collective knowledge and perspectives by involving policymakers, educators, students, and other relevant actors in developing causal loop diagrams to create more robust and inclusive analyses of fee-free higher education dynamics. Causal loop diagrams have been used in several fields to assist decision-making process. For instance, tourism scholars are increasingly advocating for the use of complexity science to understand and manage tourism governance and policymaking. Complexity science is valued because its ability to handle the unpredictable and interconnected nature of tourism, unlike traditional methods that simplify and assume linear relationships. This approach aims to provide a more accurate and comprehensive analysis of the complexities involved in policymaking ( Crabolu et al., 2023; Farsari, 2023; McDonald, 2009; I. Pappas, 2019). The application of causal loop diagrams has also been utilized to investigate policy resistance mechanisms in Southern Italy in a study that emphasizes the consequences of overlooking ambiguity in problem framing during decision-making processes. The scholars highlight that when decision-actors oversimplify the interaction space by disregarding the roles of other decision-actors or making erroneous assumptions about their mental models, it can impede the effective implementation of environmental policies ( Giordano et al., 2017).

Lastly, by linking complexity science to the study of fee-free higher education, this research builds on theoretical concepts of equity in education, resource allocation, and policy sustainability. It situates the dynamics of fee-free education within the broader discourse of education as a public good, as emphasised by scholars such as Salmi and D’Addio (2021) and Ndaba (2023). The core problem addressed by this research is the complexity of implementing fee-free higher education in South Africa and is guided by the following questions:

1. What are the key factors influencing the implementation of fee-free higher education?
2. How do these factors interact to shape the outcomes of the policy?
3. What strategies can be adopted to address the challenges of implementing fee-free higher education while ensuring its long-term sustainability?

### 1.1 Dynamics of fee-free higher education in South Africa

The debate over fee-free higher education is a prominent topic in the global education policy movement ( de Gayardon, 2018; Bray and Kwo, 2013), reflecting varying ideological, economic, and social perspectives. According to de Gayardon (2018), historically, fee-free higher education has been embraced by many European countries, including Germany and Scandinavian nations, as part of their commitment to social democracy and public welfare. As highlighted by de Gayardon (2018), these countries view higher education as a universal right that should be accessible to all, regardless of financial status. In contrast, Bray and Kwo (2013) noted that the United States has seen a more contentious debate. Proponents argue that free education reduces socioeconomic disparities and improves national competitiveness ( Bray and Kwo, 2013). However, opponents express concerns that such policies could lead to higher taxes, inefficiencies, and potential devaluation of degrees ( Bray and Kwo, 2013). The experiences of these countries provide valuable lessons for South Africa, whose socioeconomic sector presents unique challenges. As a developing nation, South Africa has one of the highest levels of income inequality globally (with a Gini coefficient of 0.67) and an unemployment rate of 32.6% ( Valodia, 2023). These figures highlight the critical role of education in addressing systemic inequality and fostering economic growth as stated earlier. However, implementing fee-free higher education in a context of limited public resources poses significant financial and administrative challenges.

In addition, Rios-Jara (2023) stressed that economically, the impact of fee-free education varies across countries. In high-income nations with robust social safety nets, the financial burden of providing free education is managed through progressive taxation and efficient public spending ( de Gayardon, 2018). Conversely, according to Rios-Jara (2023), the fiscal strain can be significant in lower-income countries, potentially diverting resources from other critical areas such as health and infrastructure. Akala (2023) also stated that fee-free education policies are often associated with positive social outcomes, including increased enrolment rates and improved educational attainment among marginalised groups. However, these policies also face challenges, such as ensuring the quality of education and managing increased demand without compromising standards ( Wangenge-Ouma, 2012). Critics argue that free education alone cannot address underlying inequalities and may require complementary measures, such as targeted support for disadvantaged students ( Varghese et al., 2023).

Research such as Ayuk & Koma (2019) has shown that South Africa’s post-apartheid educational policy has centred on equitable access to higher education. Recognising education’s crucial role in fostering social mobility and economic development, the government has implemented various measures to broaden access to tertiary education, particularly for historically marginalised communities ( Varghese et al., 2023). One of the most significant initiatives in this regard is the introduction of fee-free higher education for eligible students ( Akala, 2023). In 2015, widespread student protests erupted across South Africa under the hashtag #FeesMustFall ( Ntombana et al., 2023). These protests were triggered by the University of the Witwatersrand’s proposal to raise tuition fees for the 2016 academic year. Subsequently, following the research by Jacobs et al. (2019), similar demonstrations spread to all government-funded universities, marking a crucial moment in the country’s higher education sector. These protests catalysed a call for free higher education, driven by the recognition of the systemic failure of the South African education system to adequately serve historically marginalised and oppressed communities ( Ntombana et al., 2023). On December 16, 2017, according to the report by Wangenge-Ouma (2012), President Jacob Zuma made a surprising announcement, declaring the introduction of tuition-free education at the higher education level. Enacted in response to widespread protests and demands for accessible tertiary education, the policy aims to alleviate financial barriers and promote inclusivity within the higher education sector ( Spreen & Vally, 2006).

Akala (2023) emphasised that socioeconomic, political, and institutional factors shape the dynamics surrounding fee-free higher education in South Africa. These factors, as highlighted by Mokgotho et al. (2023), involve various stakeholders such as student representatives, The Presidency of South Africa, the Department of Higher Education and Training (DHET), the National Treasury, the Heher Commission of Inquiry into the Feasibility of making Higher Education and Training Fee-free in South Africa (Heher Commission), media outlets, and researchers. Consequently, the policymaking terrain for fee-free higher education is characterised by diverse actors with differing perspectives and interests operating within a constrained environment, where limited resources and competing demands often lead to tension and trade-offs ( Mokgotho et al., 2023). For example, the National Treasury faces the challenge of funding fee-free education while maintaining budgets for other critical sectors. Universities must balance increased student enrolment with limited infrastructure and academic resources, potentially compromising the quality of education. Student representatives play a crucial role in voicing concerns about access and inclusivity, but their demands often clash with government and institutional limitations ( Donald et al., 2018). These competing interests make decision-making a complex process, requiring careful negotiation and collaboration among stakeholders. At its core, the fee-free higher education initiative aims to rectify historical disparities in educational access and promote a more equitable society.

### 1.2 Policy framework of fee-free higher education

The policy framework governing fee-free higher education in South Africa is multifaceted ( Masutha & Motala, 2023), with the National Student Financial Aid Scheme (NSFAS) playing a central role in providing financial assistance to eligible students ( Jacobs et al., 2019). NSFAS aims to alleviate the financial burden of tertiary education by covering tuition fees, accommodation, and living expenses for qualifying students ( Ntombana et al., 2023). Additionally, ( Mokgotho et al., 2023) stressed that legislative measures have been enacted to ensure equitable access to higher education, including laws prohibiting discrimination and promoting inclusivity.

Studies conducted by the Council on Higher Education (CHE) and the Department of Higher Education and Training (DHET) offer valuable insights into the institutional mechanisms and implementation strategies of fee-free higher education ( Masutha & Motala, 2023). These studies examine the operational aspects of NSFAS, such as eligibility criteria, application processes, disbursement mechanisms, and monitoring procedures ( Mokgotho et al., 2023). Moreover, they assess the effectiveness of outreach and awareness campaigns to inform prospective students about available financial aid opportunities. Despite the intentions behind these policies and initiatives, Wangenge-Ouma (2012) highlights various challenges that hinder their effective implementation. Bureaucratic inefficiencies within NSFAS and other administrative bodies often result in delays in processing applications and disbursing funds ( Sayed & Motala, 2012), leading to student frustration and disillusionment. Furthermore, Kishun (2007) emphasised that funding constraints pose a significant barrier to expanding access to fee-free higher education, as the demand for financial aid often exceeds available resources. This issue is exacerbated by competing budgetary priorities and economic uncertainties ( Dutywa, 2022).

Furthermore, administrative complexities also contribute to the challenges of implementing fee-free higher education ( Yende & Mthombeni, 2023). The intricate requirements and documentation needed to qualify for financial aid can be daunting for students, as denoted by Wangenge-Ouma (2012), particularly those from marginalised communities with limited access to information and support services ( Ahmed & Sayed, 2009). Additionally, the decentralised nature of higher education institutions in South Africa introduces variability in implementing fee-free education policies, leading to inconsistencies and disparities in access and support ( Sayed & Soudien, 2005).

According to Sayed and Soudien (2005), to address these barriers, several actionable steps can be taken. Following the study, centralising key functions of NSFAS, such as application processing and fund disbursement, could improve efficiency and consistency. A centralised system would standardise eligibility assessments, reduce delays, and ensure equitable resource distribution across institutions. In addition to that, digitising the NSFAS application process and integrating it with national identification systems could significantly streamline administrative procedures, as stated by Ayuk and Koma (2019). This approach would simplify the application process for students, reduce the burden of manual documentation, and enhance transparency in fund allocation. Moreover, Jacobs et al. (2019) added that investing in digital infrastructure to support real-time tracking of applications and fund disbursement would allow both students and institutions to monitor progress and address issues promptly. Developing an accessible, user-friendly platform with multilingual support and targeted outreach in rural areas could further bridge the information gap for marginalised communities.

In addition to administrative reforms, Motala et al. (2023) emphasised that enhancing funding mechanisms is essential. Innovative financing models, such as public-private partnerships and the introduction of targeted education levies, could supplement government allocations and ensure the sustainability of fee-free higher education. Also, increasing funding allocations to NSFAS, paired with strict oversight to prevent mismanagement, would help meet the growing demand for financial aid ( Dutywa, 2022). Finally, Mokgotho et al. (2023) research shows that fostering stronger collaboration among policymakers, educational institutions, and other stakeholders is crucial. Establishing a centralised task force dedicated to monitoring and evaluating policy outcomes would enable continuous improvement. Regular assessments could identify implementation gaps, guide resource allocation, and ensure that the goals of equitable access and social inclusion are realised ( Salmi & D’Addio, 2021).

### 1.3 Challenges and opportunities of fee-free higher education in South Africa

Higher education serves as a vital avenue for both personal advancement and economic development ( Kioupi & Voulvoulis, 2019), playing an essential role in enhancing individual knowledge and bolstering the nation’s economy by supplying the workforce with skilled professionals ( Ndaba, 2023). A higher education qualification fosters independence and sustainability in one’s life. It contributes to the overall growth and prosperity of a developing country like South Africa, where higher education represents a significant investment in human capital ( Mlambo et al., 2018).

In light of South Africa’s triple challenges of unemployment, poverty, and inequality, investing in higher education is a strategic approach to address these pressing social issues while stimulating economic growth ( Jordaan, Van Heerden, & Jordaan, 2014). As a result, education policymakers should prioritise improving access to higher education and ensuring that the educational system equips the South African workforce with the necessary skills and competencies demanded by the contemporary job market ( Jordaan, Van Heerden, & Jordaan, 2014).

Following the definition by Salmi and D’Addio (2021), fee-free higher education is a policy designed to remove financial barriers to tertiary education, making it more accessible to a broader range of individuals. The rationale behind this policy is to increase educational access and equity ( Salmi and D’Addio, 2021), and similar to South Africa, it has been pursued by various countries such as Germany and Chile ( Rios-Jara, 2023). These nations have implemented fee-free education to enhance social mobility, reduce student debt, and support the development of a highly educated workforce ( Rios-Jara, 2023). However, while fee-free higher education represents a significant step towards promoting educational equity, its implementation has encountered several challenges ( Wangenge-Ouma, 2012). Implementing fee-free higher education has raised concerns about the long-term sustainability of funding mechanisms ( Kishun, 2007). According to Sayed & Motala (2012), while the policy aims to alleviate student financial burdens, the strain on public finances is considerable. With competing budgetary priorities and limited resources ( Sayed & Soudien, 2005), sustaining fee-free higher education programs over the long term presents a significant challenge. Moreover, economic conditions and government revenue fluctuations further exacerbate funding availability uncertainties ( Mokgotho et al., 2023).

Following the study by Ayuk & Koma (2019), addressing this challenge requires innovative financing models, including public-private partnerships and alternative revenue streams, to ensure the continued viability of fee-free higher education initiatives without compromising the quality of education or other essential services. For example, in Germany, de Gayardon (2018) denoted that progressive taxation ensures a steady stream of funds for fee-free higher education, supported by robust economic policies that prioritise education as a public good. Similarly, Norway combines government funding with modest fees for international students, generating additional revenue without burdening domestic students ( Johnstone and Marcucci, 2007). South Africa could adopt such hybrid models, integrating public-private partnerships to diversify funding sources. Partnerships with private companies, such as those seen in Australia’s “Higher Education Contribution Scheme,” can provide an innovative approach, where graduates contribute to education costs through income-contingent loans after reaching a certain income threshold ( Marks, 2009). Several studies (see e.g, Ayuk & Koma, 2019; Varghese et al. 2023; Jacobs et al. 2019 and Marire, 2017) have highlighted the role of investing in quality education and concerns of quality in the implementation of of fee-free higher education.

Lastly, this research emphasises the transformative potential of fee-free higher education in South Africa. When there is a provision for equitable access to quality education, these initiatives can break the cycle of poverty and drive socioeconomic progress. Moreover, the alignment of fee-free higher education with global development agendas such as the Sustainable Development Goals (SDGs) and the African Agenda 2063 underscores its significance in advancing inclusive growth and fostering social cohesion. The implications of this research extend beyond academia, offering valuable insights for policymakers, educators, and stakeholders seeking to address educational inequalities and promote equitable access to higher education in South Africa and beyond.

Free education has been highly debated in South Africa. However, technical grounds that supports decision-making in this regard has been less explored.

## 2. Methods

The research methodology employed in this study utilises system dynamics, specifically causal loop diagrams (CLDs), to analyse the dynamics of fee-free higher education in South Africa. Initially developed by Forrester (2009), system dynamics offers a framework to comprehend the structure and behaviour of complex systems. This methodology has found application across various domains, including project management ( Toole, 2005), education ( Faham et al., 2017), strategic planning ( Goyol & Dala, 2013), and capacity planning ( Vlachos et al., 2007). CLDs, conceptualised by Forrester (2009) depict the interconnections and feedback loops within a system. These diagrams elucidate a system’s structure and feedback mechanisms, facilitating the understanding of how behaviours manifest and enabling the development of strategies to address or mitigate them ( Abidin et al., 2017). Additionally, CLDs help ascertain the extent of interconnectivity between the focal system and other related systems, providing insights into the broader context of the issue under investigation ( Abidin et al., 2017).

In this study, CLDs are used to descriptively explore the factors shaping the dynamics of fee-free higher education policy in South Africa. The primary objective of constructing a CLD in this context is to visually illustrate causal relationships and significant feedback loops among variables within the system, thereby providing a comprehensive understanding of the policy’s implementation and impact.

The key components of the diagram, as outlined by Haraldsson (2004), include:

a. Variables: Relevant elements for describing the system.
b. Oriented arcs: Indicate causal relationships, with the + and - signs denoting positive or negative effects.
c. Positive loops (denoted as R): Represent self-reinforcing loops, where an initial disturbance leads to further change, indicating an unstable equilibrium.
d. Negative loops (denoted as B): Represent self-correcting or balancing loops, where the system seeks to return to equilibrium after a disturbance.

These elements collectively form the Causal Loop Diagram (CLD), which visually illustrates causal relationships and significant feedback loops among variables within the system ( Vlachos et al., 2007). This methodology aligns with the research aim to evaluate the dynamics of fee-free higher education policy in South Africa. By using a causal loop diagram approach, the study descriptively explores the complex interplay of various factors shaping the policy. The methodology helps to address the research gap by providing a holistic view of how different elements interact within the system, shedding light on the underlying relationships that traditional analyses, such as regression analysis or comparative case studies, might overlook ( Forrester, 2009). Traditional methods often focus on linear relationships and isolated variables. In contrast, the causal loop diagram approach captures the interconnected factors and feedback loops inherent in the system ( Haraldsson, 2004), offering a comprehensive understanding of the policy’s implementation and impact. This thorough understanding is crucial for policy-makers in developing effective strategies to enhance the policy’s outcomes and mitigate potential challenges. The subsequent section details the construction of the CLD and its application to the research.

CLDs have been constructed using two primary approaches: participatory processes or the collection of textual data through traditional qualitative methods such as interviews and document analysis. The participatory approach involves stakeholders in the analysis, facilitating a shared understanding of system complexity ( Eker et al., 2018). On the other hand, the textual data collection approach maintains high validity by utilizing multiple sources, which is particularly beneficial when participatory methods are impractical ( Eker & Zimmermann, 2016; Kim & Andersen, 2012; Yearworth & White, 2013). This paper illustrates the application of the participatory data collection method to understand the complexities in higher education public funding.

Participatory System Dynamics Modelling (SDM) approaches considers the complex, non-linear interactions among various elements that influence fee-free higher education, and integrates scientific and stakeholder knowledge. The effectiveness of this approach has been demonstrated in several recent studies ( Zomorodian et al., 2018; Santoro et al., 2019; Pagano et al., 2019; Coletta et al., 2024). Among the different participatory SDM methods, this work uses Causal Loop Diagrams (CLDs) to support collective system understanding and modeling ( Mirchi et al., 2012; Inam et al., 2015; Giordano et al., 2017; Perrone et al., 2020). CLDs help describe the complex interconnections and feedback loops affecting system dynamics, allowing for the identification of key mechanisms that produce expected co-benefits and generate trade-offs among stakeholders. CLDs were chosen for their ability to map and visualize interactions among different system components, making them accessible to non-experts and facilitating discussions among stakeholders and local experts ( Inam et al., 2015; Coletta et al, 2024).

The construction of a CLD to analyse the dynamics of fee-free higher education in South Africa is the primary objective of this research, detailed further in the subsequent section. To achieve this, Vensim Personal Learning Edition (PLE) software was selected for developing the CLD. Roos (2024) explained that Vensim PLE is widely regarded for its user-friendly interface and robust capabilities in modeling dynamic systems. It allows for efficient visualization of complex feedback loops and causal relationships, making it particularly suitable for system dynamics research. While alternative software such as Microsoft Visio, AnyLogic, and Visual Paradigm are available, Vensim PLE offers distinct advantages for this study. Unlike general-purpose diagramming tools like Microsoft Visio, Vensim PLE is specifically designed for system dynamics modeling, enabling more accurate representation of feedback mechanisms ( Rubin et al., 2019). Compared to AnyLogic, which supports advanced simulations but requires a steeper learning curve, Vensim PLE provides a balance between functionality and accessibility, making it ideal for educational and research purposes ( Roos, 2024).

### 2.1 Motivation behind methodology

Fee-free higher education policies operate within a complex socioeconomic and institutional context characterised by numerous interacting variables ( Mlambo et al., 2018). Causal loop diagrams allow for the visualisation of these intricate relationships, enabling researchers to identify feedback loops and non-linear dynamics that may not be apparent through traditional analytical methods ( Forrester, 2009). By mapping out causal relationships and feedback loops, causal loop diagrams facilitate a systemic understanding of fee-free higher education dynamics. This holistic perspective helps researchers uncover underlying patterns and mechanisms that drive policy implementation, impact, and outcomes, thereby informing more effective intervention strategies, as Abidin et al. (2017) highlighted.

Furthermore, unlike quantitative modelling techniques, such as econometric or statistical models, causal loop diagrams prioritise qualitative analysis and conceptual mapping. This approach is well-suited to exploratory research endeavours seeking to elucidate the complexities of social systems and policy environments, where precise numerical data may be limited or uncertain, as emphasised by Haraldsson (2004). Causal loop diagrams offer a participatory research tool that can engage stakeholders in the research process, similar to the research methodology ( Toole, 2005). Researchers can harness collective knowledge and perspectives by involving policymakers, educators, students, and other relevant actors in developing causal loop diagrams to create more robust and inclusive analyses of fee-free higher education dynamics.

### 2.2 Context and problem description

The context of this research revolves around the pursuit of equitable access to higher education in post-apartheid South Africa, where historical injustices and socioeconomic disparities have limited opportunities for historically marginalised communities. Despite efforts to broaden access through fee-free higher education initiatives, challenges persist, including questions about the sustainability of funding mechanisms, the quality of education, and the adequacy of support services for disadvantaged students. Addressing these challenges is imperative to ensure that fee-free higher education policies promote inclusivity and foster social mobility. Therefore, this research seeks to evaluate the dynamics of fee-free higher education in South Africa, exploring the interplay of socioeconomic, political, and institutional factors through the causal loop diagram.

### 2.3 Developing the causal loop

This section demonstrates the step by step framework of the mothod which was adopted in developing the causal loop diagram based on the System Thinking principles. The causal loop was development was carried out in three phases as illustrated in Table 1.

**Table 1.** Phases adopted for the development of the causal loop diagram (adopted from Coletta et al., 2024).

No.	Phase	Aim	Method	Expected Outcome
1	Literature review and preliminary Causal Loop Diagram (CLD) building	To build a preliminary CLD, based on the scientific knowledge and background information on the study area	• Literature review on fee-free higher education • Gathering information about the study area, for example, from reports, existing models, etc.	A preliminary CLD on the study area, based on the scientific knowledge, focused on fee-free higher education
2	Interviews with stakeholders for preliminary CLD improvement	To collect and structure stakeholder knowledge for improving the key cause-effect relationship of the preliminary CLD	• Informal discussions with stakeholders and experts • Integration of scientific and stakeholder knowledge	A CLD on fee-free higher education which integrates scientific and stakeholder knowledge
3	CLD causal structure validation	CLD causal structure validation To validate general structure and key CLD connections	Collective model testing and participatory exercises	Final structure of CLD

The first phase was to conduct a literature review and develop a preliminary causal loop diagram based on the existing knowledge of the scenario as well as the concept. This process involved an in-depth review of published articles on CLDs to understand the intricate details of the various methods that could be followed in its development as well as studying published work on fee free higher education in South Africa to identify the key variables at play. Studies show that a CLD can be co-developed with the stakeholders directly ( Inam et al., 2015; Perrone et al., 2020; Coletta et al., 2024), however, there is a significant role that independently exploring literature and developing a preliminary CLD plays in understanding the background of the dynamics of the problem. The preliminary CLD was developed to explicitly illustrate the current understanding of the relationships between the key players on the dynamics of fee-free higher education on the basis of the literature that was reviewed. The model was subsequently improved after the stakeholder and expert discussions were held.

**Figure 1.**
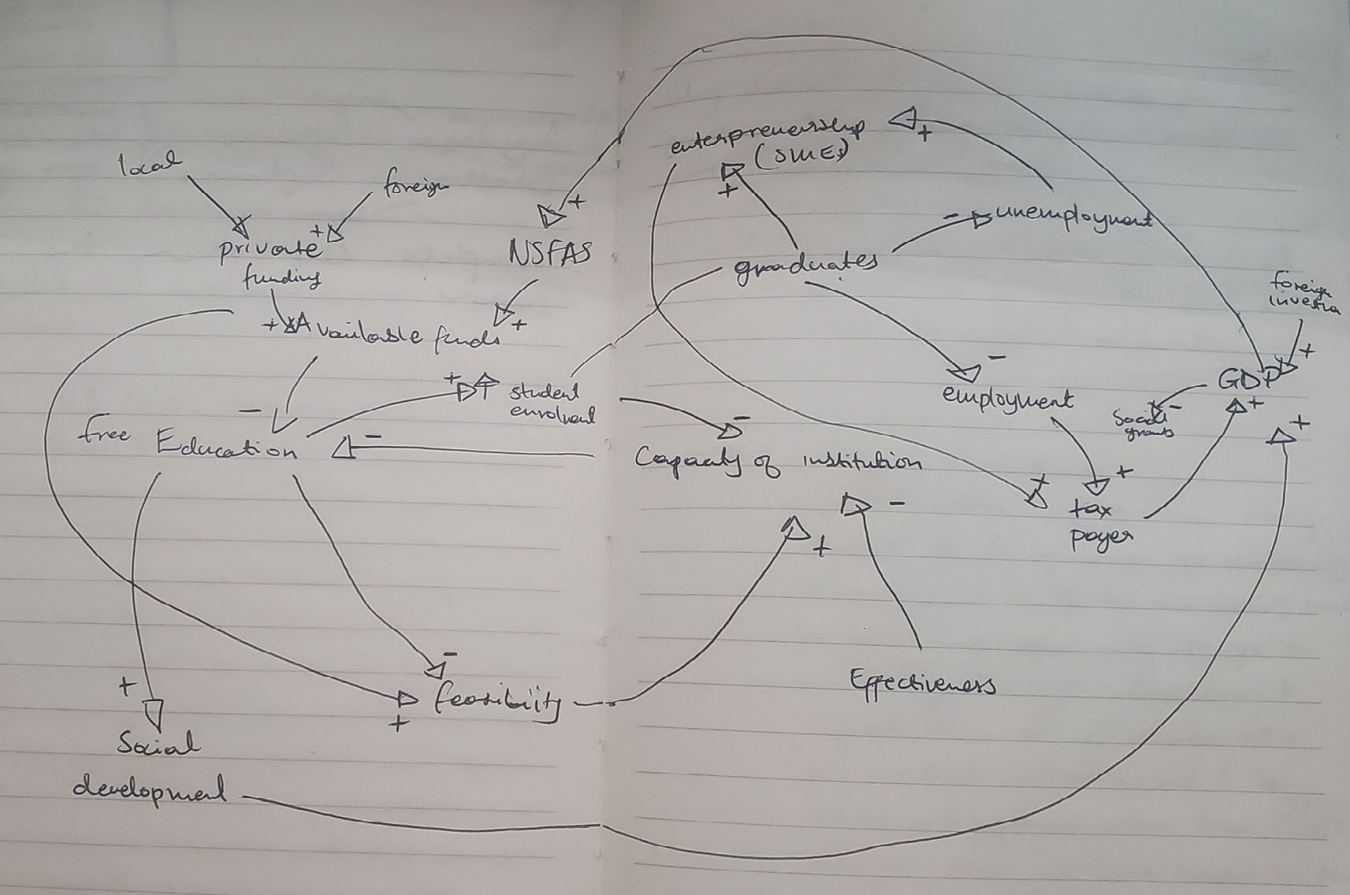
Preliminary causal loop diagram for fee-free higher education.

The second phase of developing the CLD was to involve stakeholder and expert for their input on the preliminary CLD which was developed in phase 1. This involved individual detailed informal discussions on the topic of fee-free higher education in South Africa with the aim to add value and contribute to knowledge to the CLD by addressing the system boundaries in relation to the subject, the causality (cause and effect) of the relationships, and improving the overall CLD ( Inam et al., 2015; Kotir et al., 2016; Pluchinotta et al., 2021; Salvia et al., 2021; Coletta et al., 2024). The preliminary CLD was shared with the stakeholders and experts during the discussions to ensure that the discussions are concise, remain within the context of the subject and they add value. Figure 1 shows the preliminary CLD.

The third and last phase of this study entailed the validation of the CLD by stakeholders and experts. Scholars have cited that a CLD using the participatory method can be validated by involving stakeholders and experts during various phases of the modelling process ( Mirchi et al., 2012; Coletta et al., 2024). A number of studies in different fields have as such used this to validate their work (see e.g. Bertone et al., 2019; Pagano et al., 2019; Sahin et al., 2016; Susnik et al., 2012). The another set of meetings with the stakeholders was arranged for the validation of the CLD. The variables were divided into thematic clusters and stakeholders were asked to validate relationships particularly where stakeholders expressed conflicting views in phase 2 and where there was not sufficient literature. Once the causal relationships were finalized, meetings with experts were arranged to validate the final CLD structure which incorporates both the stakeholders and experts, as well as the scientific knowledge.

## 3. Causal loop diagram for fee-free higher education in South Africa

The concept of fee-free higher education is not merely an isolated policy decision; it represents a complex system influenced by a number of interacting factors, including economic, social, and political dynamics as illustrated in Figure 1. Understanding these interdependencies is crucial in the developing the CLD. This section presents and discusses the CLD which encapsulates the key variables and their interactions within the context of fee-free higher education in South Africa. It is important to note that the CLD is a description based on the current available knowledge which can be revised and updated. It is not singular and final view of the analysed system.

### 3.1 Socioeconomic factors

**Figure 2.**
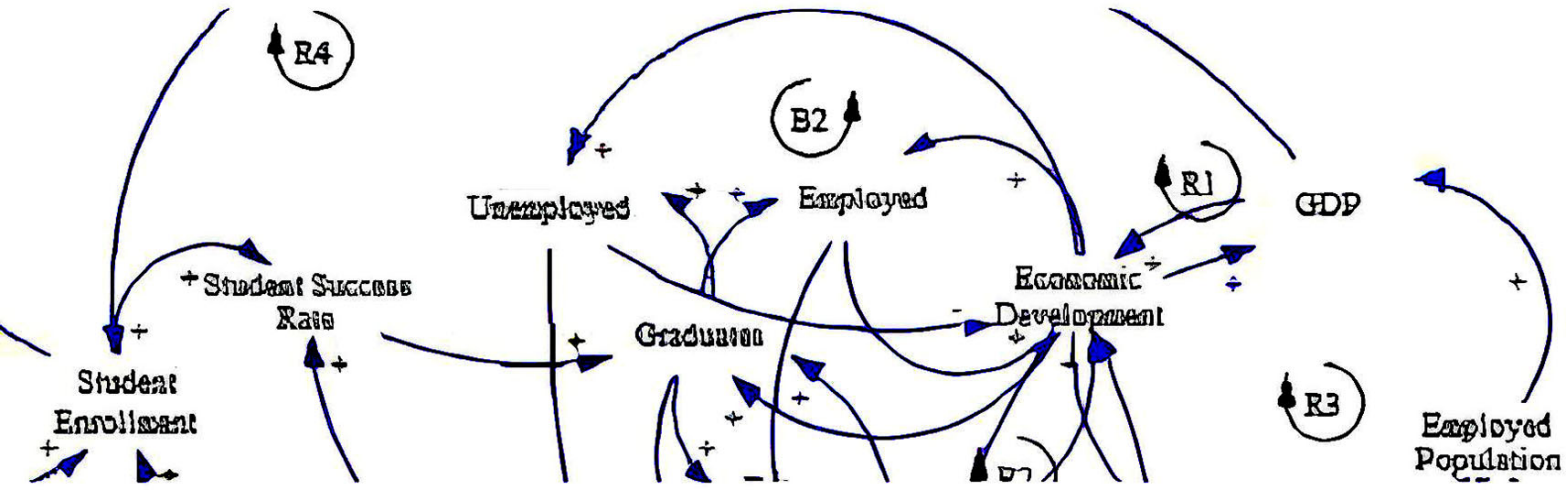
Causal relationships for social-economic factors influencing fee-free higher education.

The causal loop diagram (CLD) below ( Figure 2) aims to expound the dynamics of fee-free higher education in South Africa by examining socioeconomic factors influencing its implementation and impact. The diagram encompasses variables grouped under the socioeconomic category, including student enrolment, student success rate, unemployed individuals, graduates, employed individuals, employed population, gross domestic product (GDP), and economic development.

The CLD delineates various causal relationships among these variables with several reinforcing loops (R1, R3 & R4) and one balancing loop (B2). Foremost, student enrolment is positively related to the student success rate (see loop R4), indicating that higher enrolment rates may lead to improved student outcomes, supported by the study by Matsolo et al. (2016). This underscores the importance of ensuring access to education and the quality and support mechanisms necessary for student retention and achievement. Also, it resonates with the research emphasising the need for comprehensive student support services to enhance educational outcomes and maximise the benefits of fee-free higher education policies ( Donald et al., 2018). This positive relationship extends to graduates, suggesting that increased student success rates can result in more graduates entering the workforce.

Furthermore, graduates hold significant implications for unemployment and employment dynamics. Graduates have positive relationships with unemployed and employed individuals, implying that they can contribute to reducing unemployment or increasing employment. This highlights the potential of higher education in addressing labour market challenges and promoting socioeconomic inclusion, which aligns with the study by Vogel (2015). Conversely, the CLD also suggests that the impact of fee-free higher education on employment outcomes may vary, necessitating targeted interventions to ensure alignment between educational qualifications and labour market demands ( Donald et al., 2018).

In addition, economic development emerges as a central factor influenced by multiple variables in the CLD. It has positive relationships with GDP and the employed population (see loops R1 & R3), indicating that economic growth fosters both increased economic output and higher employment levels. This shows the importance of education in driving economic development and creating opportunities for gainful employment, as supported by Melguizo et al. (2017). Similarly, this highlights the positive correlation between investment in higher education, human capital development, and economic prosperity ( Lauder & Mayhew, 2020).

Lastly, the CLD also explains feedback loops within the system. Economic development forms a positive feedback loop with employed and unemployed individuals despite the negative relationship between unemployment and economic development (see loop B2). This suggests that sustained economic growth can lead to further employment and overall socioeconomic development ( Matsolo et al., 2016), thereby creating a balance in the system. Similarly, GDP exhibits a positive feedback loop to economic development, emphasising the reinforcing nature of economic growth through various channels. Leveraging these feedback loops effectively requires holistic approaches that address systemic barriers to education and employment, thereby maximising the socioeconomic impact of fee-free higher education initiatives ( Sneyers & De Witte, 2017).

### 3.2 Political factors

**Figure 3.**
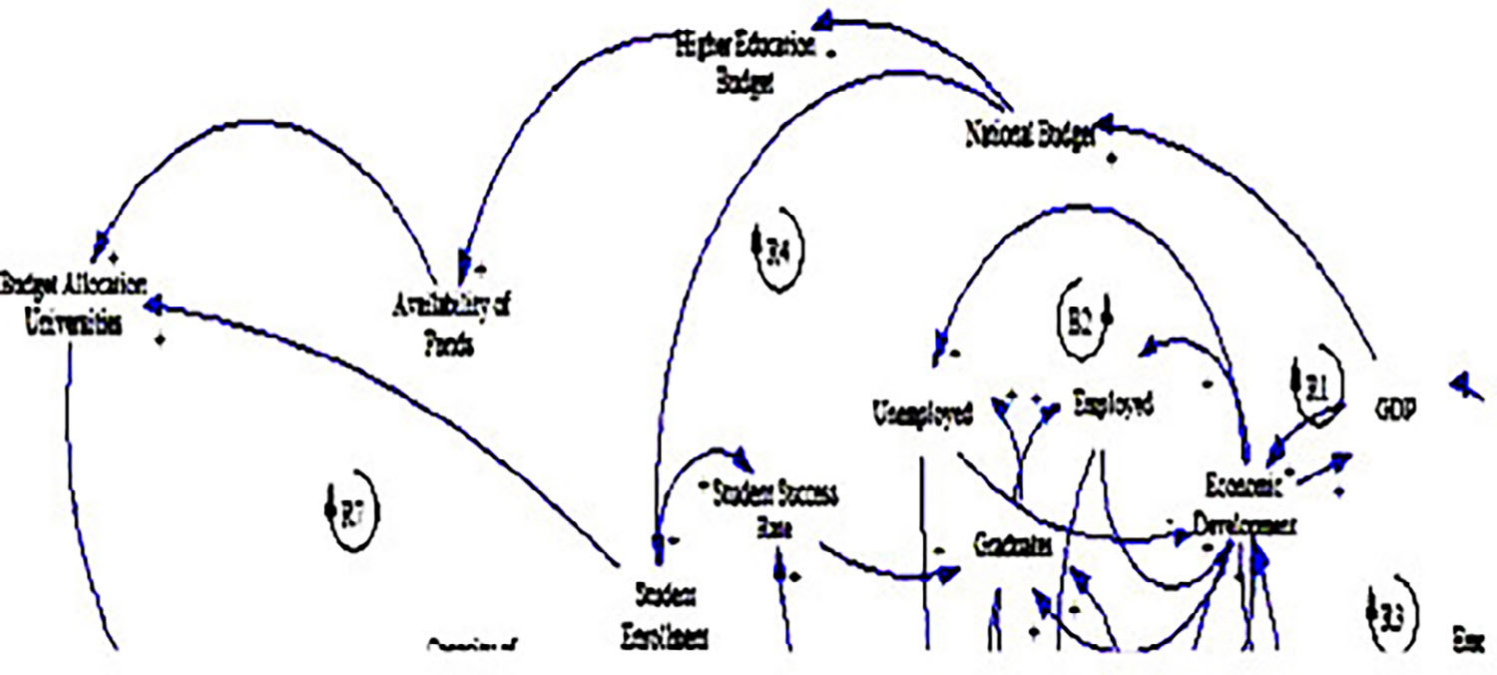
Causal relationships for political factors influencing fee-free higher education.

The causal loop diagram (CLD) below (see Figure 3) provides a comprehensive framework for understanding the complex relationships among political variables influencing fee-free higher education in South Africa. The analysis dives into the variables of budget allocation to universities, higher education budget, national budget, and availability of funds, shedding light on their interplay and implications for educational access and quality.

One of the prominent findings from the CLD is the strong positive relationship between economic growth, as measured by GDP, and government spending, represented by the national budget (see loop R1). This relationship accentuates the pivotal role of economic prosperity in enabling governments to allocate more resources to critical sectors such as education, resonating with the research by Heintz and Pollin (2008). Studies have shown that countries with higher GDP per capita tend to invest more in education, reflecting the importance of economic development in supporting public spending on social services ( Fowles, 2014). In the context of South Africa, this finding denotes the potential for sustained economic growth to bolster government support for fee-free higher education initiatives ( Tregenna, 2015).

In addition, the CLD illuminates the linkages between the national and higher education budgets, emphasising the significance of political decisions in shaping funding priorities. Government budget allocations directly influence the financial resources available for higher education ( Barro, 2013), impacting the affordability and accessibility of tertiary education for students. This finding also aligns with literature highlighting the critical role of government funding in supporting higher education institutions and ensuring equitable access to quality education ( Van Der Berg et al., 2011).

Moreover, the CLD explains the importance of adequate funding for higher education in facilitating the availability of funds for universities, aligning with the study by Oketch (2016). The positive relationship between the higher education budget and the availability of funds indicates that government investments in tertiary education directly impact the financial resources accessible to institutions. This suggests that robust government support is essential for ensuring the sustainability of fee-free higher education initiatives and enhancing the capacity of universities to accommodate students ( Brewer & McEwan, 2010).

Another significant insight emerging from the CLD is the presence of a positive feedback loop between the national budget, student enrolment, and budget allocation to universities (see loops R4 & R7). This feedback loop suggests a reinforcing cycle wherein increased government spending on education leads to higher student enrolment, prompting further budget allocations to universities. This finding underscores the potential for proactive government investment in education to stimulate demand for higher education and drive expansion and improvement in the tertiary education sector ( Faham et al., 2017). It also points to the need for strategic policy interventions to ensure sustained government support for fee-free higher education initiatives and promote inclusive and equitable tertiary education for all South Africans, as explained by Sayed & Motala (2012).

### 3.3 Institutional factors

**Figure 4.**
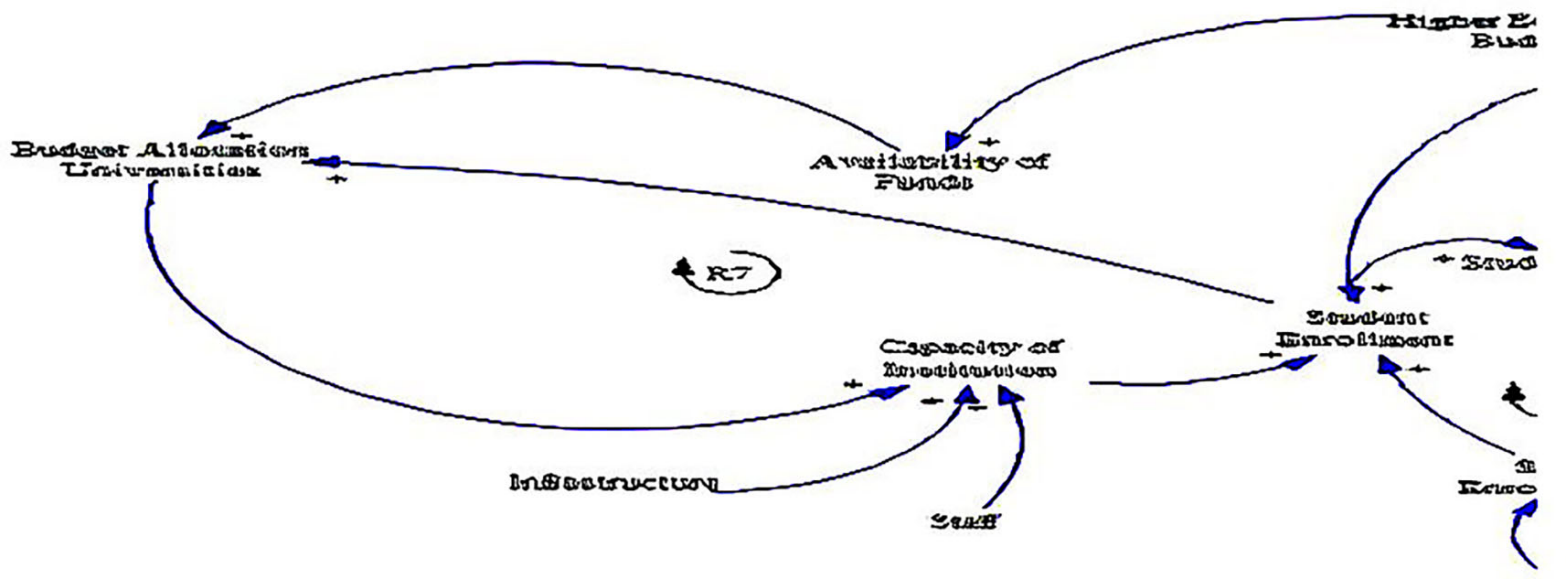
Causal relationships for institutional factors influencing fee-free higher education.

The causal loop diagram (CLD) below (see Figure 4) explores the institutional dynamics surrounding fee-free higher education in South Africa, offering a detailed examination of variables related to the capacity of institutions, infrastructure, and staff. These institutional factors play critical roles in shaping the accessibility, quality, and effectiveness of higher education initiatives, making them essential components for analysis and understanding.

One of the key insights from the CLD is the positive relationship between budget allocation to universities and the capacity of institutions (see loop R7). This relationship underlines the fundamental role of financial resources in bolstering institutional capacity, as supported by Tewari & Ilesanmi (2020). Adequate funding enables higher education institutions to invest in essential areas such as infrastructure development, faculty recruitment, and academic programs, thereby enhancing their capacity to accommodate students and deliver quality education ( Van Der Berg et al., 2011).

Likewise, the CLD points to the interconnectedness between infrastructure, staff, and the capacity of institutions. Investments in infrastructure, including buildings, laboratories, libraries, and technology, are crucial for creating conducive learning environments and supporting academic activities ( Tewari & Ilesanmi, 2020). Similarly, the availability of qualified and motivated staff members, including professors, lecturers, and support personnel, is essential for delivering high-quality teaching, research, and student support services ( Oketch, 2016).

In addition, the positive relationship between the capacity of institutions and student enrolment underlines the significance of institutional development in driving educational access and participation, resonating with Tewari and Ilesanmi (2020). Institutions with robust capacity are better equipped to accommodate larger cohorts of students, offer diverse programs, and provide adequate support services to ensure student success. By expanding institutional capacity, higher education institutions can play a pivotal role in addressing the growing demand for tertiary education and promoting inclusive access for historically marginalised and underrepresented groups ( Sayed & Soudien, 2005). This feedback loop underscores the importance of ongoing investment and strategic planning to sustainably expand institutional capacity and meet the evolving needs of students and society ( Faham et al., 2017).

**Figure 5.**
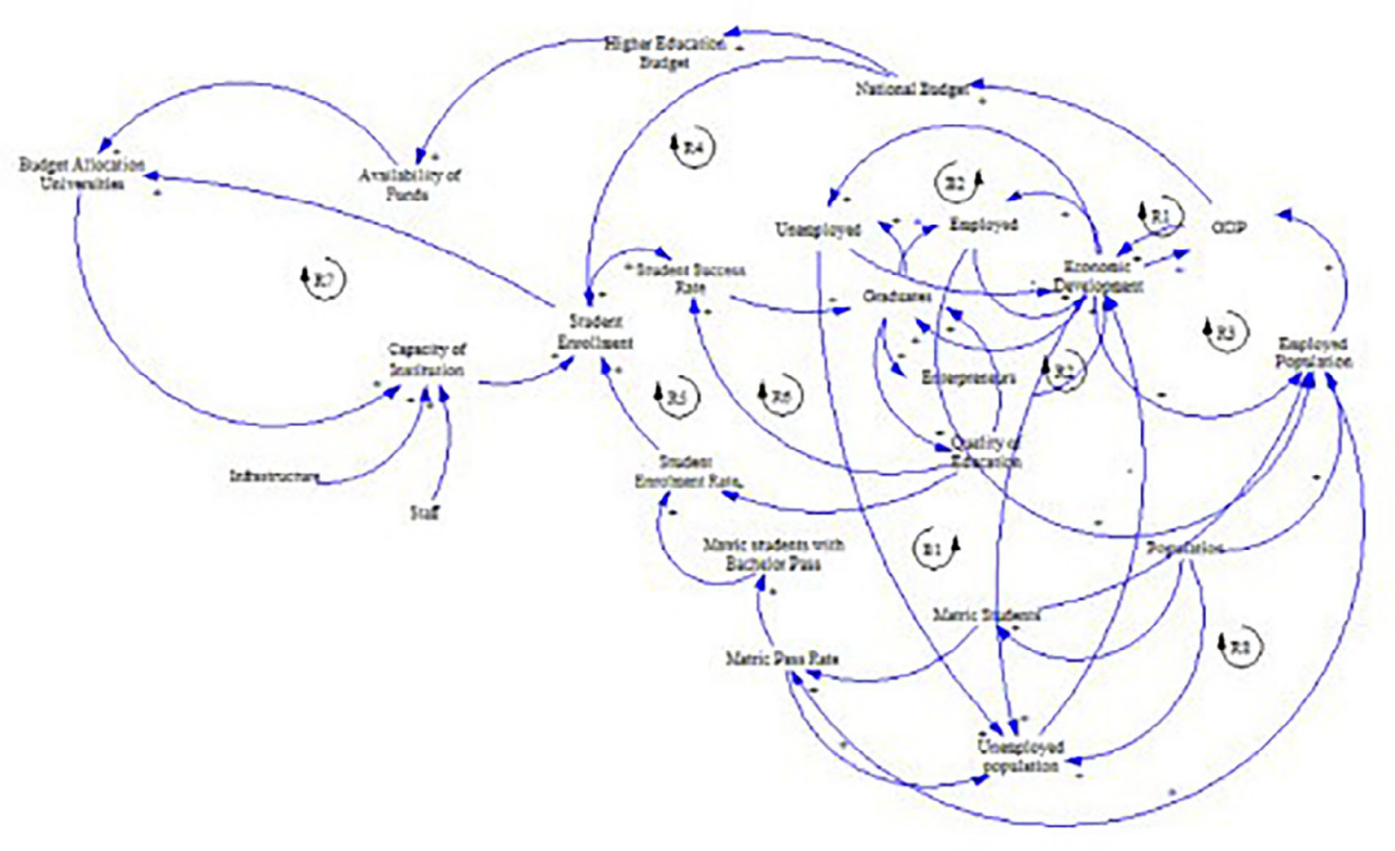
The final causal loop diagram for a fee-free higher education.

Figure 5 shows the final CLD. A list of the variables and their descriptions pertaining to this CLD are shown in Table 2 below.

**Table 2.** List of Variables and descriptions.

Variables	Description
GDP	National income
National Budget	Planned allocation of the national income
Higher Education Budget	Planned allocation to higher education
Availability of Funds	Actual money that is available for higher education
Budget Allocation Universities	The money that gets distributed to universities
Capacity of Institutions	The number of students the universities can accommodate
Student Enrolment	The number of students that register into the universities
Student Success Rate	The rate at which the students progress through the stages in the university
Graduation	Students that complete their course
Quality of Education	The effectiveness of the educational system in providing students with the knowledge, skills, and competencies necessary for personal development, economic productivity, and responsible citizenship.
Unemployment	Graduates who do not have a source of income
Employment	Graduates who are absorbed into the workplace
Entrepreneurship	Graduates who create an income for themselves
Economic Development	The national contribution made towards improving the economic well-being and quality of life of its people through growth in income, job creation, and the enhancement of infrastructure and services.
Infrastructure	The facilities and systems within the universities that enable a safe and capable learning environment
Staff	Employees of the universities that serve in various role necessary for its operation and success.
Population	The number of people in the South Africa
Employed Population	The number of employable people who are employed in South Africa
Unemployed Population	The number of employable people who are not currently employed
Matric Students	The number of students in their final year of secondary education
Matric Pass Rate	The rate at which final year secondary school students successfully complete and pass their final year of secondary school examinations.
Matric Students with Bachelor Pass	The number of secondary school students who qualify for tertiary (higher) education.

### 3.4 Implications of fee-free higher education in south africa to sustainable development goals and African Agenda 2063

The research findings on evaluating fee-free higher education in South Africa resonate strongly with several Sustainable Development Goals (SDGs) and the aspirations of the African Agenda 2063. The study emphasises the importance of equitable access to quality education, economic empowerment, and reducing socioeconomic disparities, all of which are central to the global development agenda.

The research’s alignment with ***Sustainable Development Goal 4 (SDG 4), Quality Education**,* underscores its potential in addressing global priorities for inclusive and quality education. SDG 4 is a beacon for fostering equitable access to education and promoting lifelong learning opportunities for people worldwide ( Kioupi & Voulvoulis, 2019). By emphasising the importance of enhancing student success rates and increasing the number of graduates, the research aligns closely with the core objectives of SDG 4, which seek to ensure that education is accessible, equitable, and of high quality for all individuals, regardless of background or circumstance.

Through its focus on improving student success rates, the research addresses barriers to educational attainment and strives to create a more inclusive learning environment. By identifying and addressing factors that hinder academic achievement, such as financial constraints, inadequate support services, and systemic inequalities, the research aims to promote equal access to educational opportunities and ensure that every learner has the chance to thrive, concurring with the perceptions of Jordaan, Van Heerden, and Jordaan (2014). Furthermore, by advocating for increased graduates, the research expands access to higher education and vocational training, empowering individuals with the knowledge and skills needed to contribute meaningfully to society. This aligns with SDG 4’s overarching goal of building a more educated, skilled, and empowered global population capable of driving sustainable development and fostering social progress ( Kioupi & Voulvoulis, 2019).

***Sustainable Development Goal 8 (SDG 8): Decent Work and Economic Growth.*** SDG 8 emphasises the importance of promoting sustained, inclusive, and sustainable economic growth and fostering full and productive employment for all individuals ( Frey, 2018). By equipping graduates with the skills, knowledge, and qualifications needed for employment, fee-free higher education initiatives play a crucial role in advancing the objectives of SDG 8. As graduates enter the workforce, they contribute to creating new job opportunities, innovation, and productivity enhancements, thereby driving economic growth and development, according to research by Barro (2013).

Moreover, the positive correlation between graduates and employment stresses the critical role of education in facilitating labour market participation and reducing unemployment rates ( Lauder & Mayhew, 2020). By investing in higher education and expanding access to quality educational opportunities, countries can cultivate a skilled and adaptable workforce capable of meeting the demands of evolving industries and technological advancements supported by Vogel (2015). This not only enhances individual employability and earning potential but also strengthens the overall resilience and competitiveness of the economy. In this way, fee-free higher education initiatives serve as catalysts for achieving the goals of SDG 8 by promoting inclusive economic growth, reducing inequalities, and ensuring that no one is left behind in the pursuit of decent work and sustainable livelihoods.

***Sustainable Development Goal 10 (SDG 10): Reduced Inequalities.*** SDG 10 accentuates the importance of reducing inequalities within and among countries, including income, education, and access to opportunities ( Kioupi & Voulvoulis, 2019). By addressing disparities in access to higher education, fee-free higher education initiatives play a crucial role in promoting social inclusion and levelling the playing field for individuals from marginalised communities, in line with the research by Motala et al. (2023). By removing financial barriers to education, these initiatives enable students from low-income backgrounds and historically marginalised groups to access higher education, thereby narrowing the gap between privileged and disadvantaged groups and fostering more significant social equity ( Reddy, 2004).

Moreover, the research findings highlight the potential of fee-free higher education initiatives to improve outcomes for historically marginalised groups, such as reducing dropout rates, increasing graduation rates, and enhancing employment opportunities, which also correlates with the study of Sneyers & De Witte (2017). By providing students from marginalised backgrounds with the resources and support needed to succeed in higher education, these initiatives contribute to breaking the cycle of intergenerational poverty and inequality ( Brewer & McEwan, 2010). Furthermore, by promoting diversity and inclusivity within higher education institutions, fee-free higher education initiatives create opportunities for individuals from diverse backgrounds to thrive and contribute to society ( Ndaba, 2023). In this way, free higher education initiatives align with the objectives of SDG 10 by addressing systemic inequalities and promoting a more equitable and inclusive society for all members.

***African Agenda 2063:*** The research findings are closely aligned with the aspirations outlined in the African Agenda 2063, a strategic framework for the socioeconomic transformation of the African continent. One of the key pillars of the African Agenda 2063 is human capital development, which emphasises the importance of investing in education, skills development, and lifelong learning to unlock the potential of Africa’s youth population ( Addaney, 2018). By investing in fee-free higher education initiatives, South Africa can contribute significantly to advancing this objective by equipping its youth with the knowledge, skills, and qualifications needed to drive economic growth, innovation, and sustainable development across the continent ( DeGhetto et al., 2016). Through access to quality higher education, South African youth can become catalysts for positive change, leveraging their expertise and talents to address pressing challenges and seize emerging opportunities in key sectors such as technology, healthcare, and agriculture, corroborating with the research by Reddy (2004).

Furthermore, the research’s focus on fee-free higher education aligns with the goal of economic transformation outlined in the African Agenda 2063. By expanding access to higher education and promoting skills development, South Africa can foster a more dynamic and competitive economy, generating employment, reducing poverty, and promoting shared prosperity for all citizens ( Mhangara et al., 2019). By nurturing a skilled and entrepreneurial workforce, fee-free higher education initiatives lay the foundation for sustainable economic growth and inclusive development, aligning with the vision of the African Agenda 2063 to create “the Africa We Want” - a continent characterised by shared prosperity, peace, and sustainable development ( Nicolaides, 2011). In this way, South Africa’s investment in fee-free higher education benefits its citizens. It contributes to advancing the broader goals of socioeconomic transformation and continental integration outlined in the African Agenda 2063 ( Mhangara et al., 2019).

## 4. Conclusion

The research evaluated the dynamics of fee-free higher education in South Africa through a causal loop diagram (CLD) approach, to analyse the socio-economic, political, and institutional factors influencing the implementation of the policy. The study situated fee-free higher education within the historical context of apartheid-era inequalities, during which black South Africans were systematically denied access to quality education. While fee-free education aimed to address systemic inequities by removing financial barriers, the study identified significant implementation challenges, including limited funding, administrative inefficiencies within NSFAS, and inconsistent policy application across institutions. These issues perpetuate disparities in access and quality, with financial sustainability emerging as a critical concern.

To address these gaps, the research recommended reforms such as centralising NSFAS operations, digitising application processes, and integrating them with national identification systems to improve efficiency. Exploring public-private partnerships for sustainable funding was also proposed, aligning education outcomes with labour market needs. In addition, the findings of this study aligned with global development agendas, including SDGs 4, 8 and 10, and the African Agenda 2063, highlighting fee-free higher education’s potential to increase access to education, reduce inequalities, and promote economic growth. Lastly, the implications of this research extend beyond academia, offering actionable recommendations for policymakers including strengthening monitoring frameworks, prioritising investments in under-resourced institutions, and forming a task force to align fee-free education with broader socio-economic goals. These measures can help South Africa maximise the transforming potential of fee-free higher education, ensuring it fulfils its promise to create a more equitable and prosperous society.

### 4.1 Limitations and future research directions

While this study provides significant insights into implementing fee-free higher education in South Africa, it has several limitations. The research primarily relied on secondary data and causal loop diagram analysis, which may not fully capture the keen perspectives of policymakers and other stakeholders involved in the policy’s implementation. Future research should incorporate interviews and other qualitative methodologies to add more data and policymakers’ views on the main problems and perceptions that authorities and policymakers face implementing the policy. This approach would provide a more comprehensive understanding of the challenges and practical realities encountered during the policy implementation.

Secondly, the study’s focus was predominantly on the structural and systemic aspects of the policy, potentially overlooking individual experiences and localised impacts. Future research should address these gaps by exploring the lived experiences of students, educators, and administrators within the fee-free higher education framework. Lastly, longitudinal studies could offer deeper insights into the long-term effects and sustainability of the policy. Future research should expand the geographical scope to include comparisons with other countries implementing similar policies to enrich the understanding of best practices and potential pitfalls.

### Ethics and consent

Ethical approval and consent were not required.

## Data Availability

No data are associated with this article. No extended data was used.
