# Supplementary material for: Evaluating the dynamics of fee-free higher education in South Africa: a causal loop diagram approach 
Source: F1000Res. 2025 Jan 20;13:780. Originally published 2024 Jul 9. [Version 3] doi: 10.12688/f1000research.152478.3 (PMC11775444; doi:10.12688/f1000research.152478.3)
Supplement: Peer review history

# Reviewer report: Reviewer response for version 3

**DOI:** 10.5256/f1000research.176926.r360903

**Published:** 2025-01-28

**Authors:** Fayyaz Hussain Qureshi

**Affiliations:** 1 Oxford Business College, Oxford, England, UK

I have seen most of my comments have been addressed. I do not have more comments on my side.

Is the work clearly and accurately presented and does it cite the current literature?

Partly

If applicable, is the statistical analysis and its interpretation appropriate?

Partly

Are all the source data underlying the results available to ensure full reproducibility?

Partly

Is the study design appropriate and is the work technically sound?

Partly

Are the conclusions drawn adequately supported by the results?

Partly

Are sufficient details of methods and analysis provided to allow replication by others?

Partly

Reviewer Expertise:

NA

I confirm that I have read this submission and believe that I have an appropriate level of expertise to confirm that it is of an acceptable scientific standard.

# Reviewer report: Reviewer response for version 2

**DOI:** 10.5256/f1000research.170454.r331942

**Published:** 2024-11-28

**Authors:** Sakhiseni Yende

**Affiliations:** 1 University of the Western Cape, Cape Town, South Africa

Dear Journal Editor- I now had an opportunity to review the article titled: Evaluating the dynamics of fee-free higher education in South Africa: a causal loop diagram approach

**Summary of the Article**

 The article explores the dynamics of implementing fee-free higher education in South Africa using a causal loop diagram approach. The study examines the systemic interactions between social, economic, and policy variables that influence the feasibility and sustainability of fee-free education. The authors have presented a novel methodology by applying systems thinking to analyse the complex interdependencies in the higher education sector, contributing valuable insights to the ongoing discourse on equitable access to education. 

**Evaluation of the Article**

*Significance and Contribution*

 The article addresses a critical topic with national and global implications. It sheds light on a pressing issue in South African higher education and provides an innovative analytical framework. The application of causal loop diagrams adds a fresh perspective to the literature.

**Strengths**

- The topic is timely and relevant.
- The methodology is well-suited for understanding complex systems.
- The discussion highlights practical implications for policymakers.

**Weaknesses and Areas for Improvement**

- **Clarity of Objectives**: The research objectives could be articulated more explicitly in the introduction to help readers understand the study’s scope and goals.
- **Literature Review**: While the article references key studies, there is a need to expand the literature review to include recent and diverse perspectives on fee-free education, particularly from developing countries with similar socio-economic contexts.
- **Methodological Rigour**: The causal loop diagram methodology is novel.
- **Limitations**: The article does not sufficiently acknowledge the limitations of the study or the causal loop diagram approach, which might oversimplify certain real-world complexities.
- **Recommendations**: While the discussion is insightful, the recommendations for policymakers could be expanded with concrete, actionable strategies supported by the findings. 

Is the work clearly and accurately presented and does it cite the current literature?

Yes

If applicable, is the statistical analysis and its interpretation appropriate?

Yes

Are all the source data underlying the results available to ensure full reproducibility?

Yes

Is the study design appropriate and is the work technically sound?

Yes

Are the conclusions drawn adequately supported by the results?

Yes

Are sufficient details of methods and analysis provided to allow replication by others?

Yes

Reviewer Expertise:

Musicology, ethnomusicology, music education, higher education; African music, gospel music, opera, political sciences, and education

I confirm that I have read this submission and believe that I have an appropriate level of expertise to confirm that it is of an acceptable scientific standard.

# Reviewer report: Reviewer response for version 2

**DOI:** 10.5256/f1000research.170454.r335032

**Published:** 2024-11-14

**Authors:** Fayyaz Hussain Qureshi

**Affiliations:** 1 Oxford Business College, Oxford, England, UK

**Evaluating the dynamics of fee-free higher education in South Africa: a causal loop diagram approach**

**Comments**

**Abstract**

1. **Clarity and Scope: **The abstract provides a clear overview of the study’s purpose, methodology, and key findings. However, it could benefit from a more explicit statement on the specific research questions or objectives. Clarifying the main issues that the research seeks to address within fee-free higher education in South Africa would strengthen the background section. Please use the full form and abbreviation (causal loop diagram CLD), abbreviation is missing under the heading of Background.
2. **Methodology**: The mention of a participatory approach and causal loop diagram (CLD) is informative, but additional context on the methodology would improve clarity. Briefly elaborating on why the causal loop diagram approach was chosen and how it uniquely contributes to understanding the complexities of fee-free education would enhance the reader’s understanding of the research’s significance.
3. **Results and Implications**: The abstract summarizes findings effectively; however, the results section would be strengthened by including more specific examples of the identified causal relationships. This would make the findings more tangible for readers. Additionally, indicating the broader implications of these findings for policy-making, particularly concerning the socioeconomic and political landscape in South Africa, would underscore the study’s relevance.
4. **Conclusion**: The conclusion effectively highlights the need for reforms but could be more definitive about the recommended actions. Adding a sentence on potential reform areas or the types of administrative changes that could address identified issues would provide a more actionable insight for readers.
5. **Language and Flow**: The abstract is well-written overall, though minor revisions for flow and coherence would improve readability. For instance, rephrasing "the CLD has explicitly mapped out the causal relationships which play a fundamental role" to a more concise statement would increase clarity.
6. **Completeness**: Consider including a brief mention of limitations or future research directions to provide a fuller picture of the study's scope and potential next steps.

**Overall**: This abstract presents a satisfactory foundation, but with minor additions to the methodology, examples in the results, and a more actionable conclusion, it would be more comprehensive and impactful.

**Introduction**

 The introduction provides a comprehensive background, detailing the significance of higher education in South Africa, its historical context, and the policy challenges related to fee-free education. However, the section could benefit from a clearer statement of the problem to succinctly define the core issues motivating the study. This would allow readers to more immediately understand the central focus of the research.

**Theoretical Framework**: The introduction would be enhanced by explicitly linking the discussion of complexity science and policy analysis to a broader theoretical framework or set of concepts guiding the research. This could provide a stronger foundation for understanding how CLD fits within the existing body of literature on educational policy analysis.

**Literature Review and Relevance**: The review of related studies, such as the impacts of fee-free higher education and the socioeconomic context of South Africa, is satisfactory. However, some of the cited studies could be more closely tied to the specific dynamics of fee-free education rather than broader socioeconomic issues. Streamlining the literature review to focus on studies directly related to fee-free education and similar CLD applications would enhance its relevance.

**Dynamics of Fee-Free Higher Education in South Africa**

 The section effectively situates the debate on fee-free higher education within a global context, drawing useful comparisons between South Africa and other countries. However, it could be enhanced by linking these international examples more directly to South Africa’s specific socioeconomic context, helping readers understand how South Africa’s unique historical and economic landscape influences its approach.

 The mention of various stakeholders (e.g., DHET, National Treasury, student representatives) is valuable, yet the role and influence of these stakeholders in shaping policy outcomes could be elaborated further. Discussing how competing interests and constraints influence the decision-making process could provide a richer understanding of the challenges faced in implementing fee-free education.

**Policy framework of fee-free higher education**

 The section provides a detailed overview of the key components of South Africa's fee-free higher education policy, notably the role of NSFAS and legislative measures aimed at promoting inclusivity. This foundation is valuable for understanding the operational framework of fee-free education.

 The section could benefit from a more targeted discussion of how to address specific barriers, such as administrative decentralization. For example, suggestions for centralizing certain NSFAS functions or digitizing application processes could add actionable insights to the policy framework.

**Challenges and opportunities of fee-free higher education in South Africa**

 The section effectively presents the dual nature of fee-free higher education, outlining both its potential to drive social and economic progress and the challenges it poses in terms of funding and sustainability. This balanced approach is commendable and captures the policy's complexities well. However, strengthening the analysis with more concrete examples of successful funding models and quality-maintenance strategies would add depth, making the discussion even more valuable for stakeholders involved in education policy planning.

**Methods**

 The section provides a thorough overview of the system dynamics methodology and its specific application using Causal Loop Diagrams (CLDs). The methodology section demonstrates a well-considered approach, using system dynamics to address complex, interconnected factors in fee-free higher education policy. Adding more context on specific stakeholder engagement and justifying software selection would strengthen the methodological transparency and comprehensiveness for readers and reviewers.

 The motivation for selecting causal loop diagrams (CLDs) is well-founded, particularly given the complex and interconnected nature of fee-free higher education policies.

**Causal loop diagram for fee-free higher education in South Africa**

**Suggestions for Improvement**:

- **Clarify Key Variables**: Provide a brief description of the key variables involved in the CLD (e.g., funding, student access, quality of education, economic constraints, political support).
- **Expand on the Revision Process**: Elaborate on how the CLD will be updated with new information or evolving policy conditions.
- **Elaborate on the Diagram's Construction**: Include more information on the methods used to develop the CLD and how stakeholder input was incorporated (if applicable).
- **Explain the Diagram’s Dynamics**: Highlight the significant feedback loops and interactions within the diagram to give readers a deeper understanding of the key dynamics at play.

**Conclusion**

 The conclusion effectively summarizes the key elements of the research, highlighting the analysis of the interplay of socio-economic, political, and institutional factors in the context of fee-free higher education in South Africa.

**Suggestions for Improvement:**

- **Clarify Historical Context**: Provide further detail on how apartheid-era inequalities directly affect fee-free higher education today, and how the policy attempts to address these challenges.
- **Expand on Key Findings**: Include more specific findings from the research, particularly regarding the barriers to effective implementation.
- **Reforms and Solutions**: Briefly mention potential reforms or solutions to address the gaps in policy implementation, particularly concerning financial sustainability and administrative efficiency.
- **Connection to Global Agendas**: Specify how the findings align with specific SDGs or African Agenda 2063 goals, and discuss the potential impact of the policy on these frameworks.
- **Actionable Recommendations**: Provide concrete recommendations for policymakers or stakeholders based on the research findings.

Is the work clearly and accurately presented and does it cite the current literature?

Partly

If applicable, is the statistical analysis and its interpretation appropriate?

Partly

Are all the source data underlying the results available to ensure full reproducibility?

Partly

Is the study design appropriate and is the work technically sound?

Partly

Are the conclusions drawn adequately supported by the results?

Partly

Are sufficient details of methods and analysis provided to allow replication by others?

Partly

Reviewer Expertise:

Higher Education

I confirm that I have read this submission and believe that I have an appropriate level of expertise to confirm that it is of an acceptable scientific standard, however I have significant reservations, as outlined above.

# Reviewer report: Reviewer response for version 1

**DOI:** 10.5256/f1000research.167243.r302491

**Published:** 2024-08-02

**Authors:** Héctor Ríos-Jara

**Affiliations:** 1 Postdoctoral researcher, Universidad Central de Chile, Santiago, Chile

Dear Authors, 

 I am pleased to read the article "Evaluating the dynamics of fee-free higher education in South Africa: a causal loop diagram approach." The article aims to map out causal relationships and feedback loops. Causal loop diagrams facilitate a systemic understanding of fee-free higher education dynamics. This holistic perspective helps researchers uncover underlying patterns and mechanisms that drive policy implementation, impact, and outcomes. 

 The paper picks a relevant topic and case regarding the rise of new free education policies. The paper also offers a good summary of the policy, history and current dilemmas based on the literature. The methodology is innovative in the study of policies. However, I think the paper is not ready for indexing, and it lacks internal consistency. The methodology is too descriptive and does not allow it to fulfil the aims declared in the paper: "uncover underlying patterns and mechanisms that drive policy implementation, impact, and outcomes". 

 The paper is also disconnected from the international debate on free education and the implementation challenges, despite South Africa being one the most relevant cases of new free education policies in the XXI century. 

 The case and information are valuable, but in my view, the paper requires a redefinition of the aims so the method and results match the paper's aims. If the authors decide to keep the aims, I recommend complementing current data with new information, such as interviews with policymakers or quantitative data. The main reason for this suggestion is that the results are too descriptive and do not support many claims described by the author in the results and analysis.

 Rebuilding the fluxes and procedures involved in the implementation of the policy tells a lot about how the policy works but not necessarily the problems, outcomes and challenges, which are the main aims of the paper. In addition, the paper can add more value if the authors introduce the debate of free education policies and their difficulties and frame South Africa as a case in the broader debate on the challenges of new free education policies. I hope my comments can improve the paper and not discourage the authors from publishing it because the case and information have a lot of potential but require major changes in its frame, narrative and methodology.

 I hope the authors consider these comments valuable and do not discourage them from developing the article because the paper has great potential. The authors picked a relevant case and problem for the international research community interested in higher education funding policies, particularly in free education policies. See below for more details and suggestions. 

**1) Is the work clearly and accurately presented, and does it cite the current literature? (Partly)**

 The paper does not address a wider debate on free education policies in international literature, particularly for the new generation of free education policies and their shared challenges. Consequently, it remains descriptive and focuses only on the South African case, which is relevant to a country, but also a case of policy innovation. See some references to check and include. 

 To improve the link with international debate on free education I recommend the following papers:

 Ariane de Gayardon (2019 [ Ref - 1] ). There is no such thing as free higher education: A global perspective on the (many) realities of free systems. *Higher Education Policy*, *32*(3), 485-505.

 H. Rios-Jara (2023 [Ref - 2]). The free education policy in Chile: Between transforming and maintaining neoliberalism. *South African Journal of Higher Education*, *37*(6), 112-130.

 Angelica Buendia et al. (2024 [Ref - 3]). El mito de la gratuidad en la educación superior mexicana: Acercamientos a la comprensión de un cambio de política. *Education Policy Analysis Archives*, *32*.

**2) Is the study design appropriate, and is the work technically sound? (no)**

 The paper does not explicitly state a research question or a specific aim, so it is hard to see how the methodology feeds the research question. The analysis regarding the methodology and mixed impacts with policy implementation challenges is also ambiguous, going beyond the evidence.

 It is not clear what problems free education policies face. The literature review gave a good overview, but the paper might clarify the specific problems that the policy faces. I suggest linking these problems with the international debate and similar issues that other free education policies face. Clarify the contribution and purpose of the causal loop diagrams and how the methodology is connected to the research question. Clarifying a specific research question will also improve the consistency of the paper.

**3) Are sufficient details of methods and analysis provided to allow replication by others? (partly)**

 The description of the methodology is good. But the rationale behind it requires more details. It is not clear how the methodology helps to explore the research gap that the paper aims to address. Beyond the general uses of the methods, the methodology section should make explicit why and how this method helps to address the research gap.

 For example, "Furthermore, unlike quantitative modelling techniques, such as econometric or statistical models, causal loop diagrams prioritise qualitative analysis and conceptual mapping. This approach is well-suited to exploratory research endeavours seeking to elucidate the complexities of social systems and policy environments, where precise numerical data may be limited or uncertain, as emphasised by Haraldsson (2004)." The argument is too general and is not connected to the research gap and policy implementation challenges.  

 In addition, the distinction between economic, institutional, and economic factors is vague, and there is no specific definition of the variables included in each factor. It is relevant to unpack the "dynamics of fee-free higher education in South Africa" concept and clarify the research gap, how the article contributes to its exploration, and the variables that the article considered.

**4) Are all the source data underlying the results available to ensure full reproducibility? (Partly)**

 The results presented do not provide evidence for all the analyses described in the paper. The analyses use quantitative jargon that does match the methods selected.

 In addition, the figures in section 3 are visually confusing. They do not help to explore the policy. I suggest developing a more stylised version of the figures and a better description of them. The integration of data and analysis needs to be more precise and better grounded in the information provided. If the data suggests further hypotheses, the author needs to make explicit that some statements are hypotheses, not data descriptions or analysis based on data.

 For example, "One of the prominent findings from the CLD is the strong positive relationship between economic growth, as measured by GDP, and government spending, represented by the national budget (see loop R1)." The language used in this sentence came from quantitative analysis. The figure does not show a "strong positive relationship". Moreover, the figure reveals the institutional flux of decisions and factors that attached the policy to national spending. Still, more information about policymakers' decisions and dilemmas must be available. The article closes this relevant information with general assumptions based on the literature that do not integrate data from the case.

 Another example "the CLD illuminates the linkages between the national and higher education budgets, emphasising the significance of political decisions in shaping funding priorities. Government budget allocations directly influence the financial resources available for higher education ( Barro, 2013), impacting the affordability and accessibility of tertiary education for students. This finding also aligns with literature highlighting the critical role of government funding in supporting higher education institutions and ensuring equitable access to quality education ( Van Der Berg et al., 2011)" The figure describes the procedural link between the national budget and the policy but does not open the dilemmas face by government. One of the main critiques of free education policies is its cost and financial sustainability, a topic of concern in South Africa, which, however, the paper does not address properly cause it does not add relevant information about how the policy is funded and what the dilemmas that policymakers and authorities face. Showing the links gives enough information about what is happening inside and how the actor deals with those problems.

 Another example. The analysis goes beyond the data. "Another significant insight emerging from the CLD is the presence of a positive feedback loop between the national budget, student enrolment, and budget allocation to universities (see loops R4 & R7). This feedback loop suggests a reinforcing cycle wherein increased government spending on education leads to higher student enrolment, prompting further budget allocations to universities." Here, the data show a link between them, but the paper does not provide any other evidence to show that rising government investment leads to more enrolments. It is unclear if the figure shows an expectation of policymakers or a link that might be considered a further hypothesis.  

 The analysis of the policy based on the Sustainable Development Goal 4 (SDG 4), Quality Education, came late in the article and it needs to be clarified why they are relevant for the paper. The introduction describes the policy as a consequence of protests and the aims to improve access and equality, which might be close to the SDG, but the policy was not introduced to achieve SDG.

**5) Are the conclusions drawn adequately supported by the results?**

 Unfortunately, the conclusion goes beyond what the paper shows. For example, "Through an analysis of historical contexts, policy frameworks, and institutional mechanisms, the study has illuminated the complexities surrounding access to tertiary education in the country. As the barriers posed by bureaucratic inefficiencies, funding constraints, and administrative complexities were outlined, the study underscored the urgent need for systemic reforms to enhance the accessibility and effectiveness of fee-free higher education initiatives".

 The paper does not summarise the dilemmas or challenges of free education implementation in South Africa or its problems. The methodology used helps to map and understand how the policy works, which is a relevant result, but it does not add any further information about the pressing dilemmas that policymakers face or what kind of problems and solutions policymakers are debating.

 I see two possible solutions here. First, change the paper's aims and frame and make it only descriptive, focusing on understanding how the policy works. The main contribution will be showing how the policy has been implemented. Second, to add more data and policymakers' views on the main problems and perceptions that authorities and policymakers face implementing the policy. This would require adding interviews and other methodologies to the research, but the paper will be more relevant, especially if the authors decide to frame it in the context of the international debate on the rise of new free education policies.

Is the work clearly and accurately presented and does it cite the current literature?

Partly

If applicable, is the statistical analysis and its interpretation appropriate?

Not applicable

Are all the source data underlying the results available to ensure full reproducibility?

Partly

Is the study design appropriate and is the work technically sound?

No

Are the conclusions drawn adequately supported by the results?

No

Are sufficient details of methods and analysis provided to allow replication by others?

Partly

Reviewer Expertise:

Higher education funding policy, social policy, political economy

I confirm that I have read this submission and believe that I have an appropriate level of expertise to state that I do not consider it to be of an acceptable scientific standard, for reasons outlined above.

## References

[1] : There is No Such Thing as Free Higher Education: A Global Perspective on the (Many) Realities of Free Systems. *Higher Education Policy* .2019;32(3) : 10.1057/s41307-018-0095-7 485-505 10.1057/s41307-018-0095-7

[2] : The free education policy in Chile: Between transforming and maintaining neoliberalism. *South African Journal of Higher Education* .2023;37(6) :112-130 Reference source

[3] : El mito de la gratuidad en la educación superior mexicana: Acercamientos a la comprensión de un cambio de política. *Education Policy Analysis Archives* .2024;32: 10.14507/epaa.32.8501 10.14507/epaa.32.8501
